# Construction of mitochondrial signature (MS) for the prognosis of ovarian cancer

**DOI:** 10.1007/s12672-025-02892-7

**Published:** 2025-07-22

**Authors:** Miao Ao, You Wu, Kunyu Wang, Haixia Luo, Wei Mao, Anqi Zhao, Xiaomeng Su, Yan Song, Bin Li

**Affiliations:** 1 A Department of Gynecology Oncology, National Cancer Center/National Clinical Research Center for Cancer/Cancer Hospital, Chinese Academy of Medical Sciences and Peking Union Medical College, Beijing, China; 2 A Department of Pathology, National Cancer Center/National Clinical Research Center for Cancer/Cancer Hospital, Chinese Academy of Medical Sciences and Peking Union Medical College, Beijing, China; 3 Obstetrics and Gynecology Department, Beijing Huairou Maternal and Child Health Care Hospital, Beijing, China

**Keywords:** Ovarian cancer, Tumor microenvironment, Immunotherapy, Biomarker

## Abstract

**Background:**

Ovarian cancer (OV) continues to be the most lethal type of gynecological cancer with a poor prognosis. During tumorigenesis and cancer advancement, mitochondria are key players in energy metabolism. This study focuses on exploring the mitochondria-related genes for the prognosis of OV.

**Methods:**

RNA expression profiles and single-cell data were acquired from The Cancer Genome Atlas (TCGA), International Cancer Genome Consortium (ICGC), and Gene Expression Omnibus databases for screening and validating mitochondria-related differentially expressed genes (DEGs). After univariate Cox analysis, prognostic genes were carried out for modeling mitochondria signature (MS) based on 101 combinations of 10 machine learning algorithms. Functional enrichment analysis was performed on this prognostic gene set. Immune infiltration analysis was performed between MS groups. Validation for the prognostic model gene OAT was performed to identify the prognostic significance, combined with *in vitro* experiments to explore its expressions in OV cells. qRT-PCR assay was performed to examine the expression of OAT in human ovarian cancer cell samples and normal ovarian epithelial cells.

**Results:**

A total of 21 prognostic mitochondria-related DEGs were identified for reliably constructing the model MS with excellent prognostic performance in OV. GO and KEGG analysis confirmed these genes were enriched in the generation of precursor metabolites and energy. It illustrated more lymphocyte infiltration in the high MS group than low MS group. OAT served as a novel biomarker for OV patients, showing poor survival in OV patients with high expression of OAT. qPCR assays confirmed its significantly high expression in human ovary cancer cell lines.

**Conclusions:**

The MS offers tailored risk evaluations and immunotherapy treatments for each OV patient. MS model gene OAT has been recognized as a new oncogene for OV linked to immune escape.

**Supplementary Information:**

The online version contains supplementary material available at 10.1007/s12672-025-02892-7.

## Introduction

Ovarian cancer (OV) ranks as the eighth leading cause of cancer-related deaths among women globally [1]. The absence of effective screening methods makes early detection challenging, resulting in a high mortality rate. Three-quarters of OV cases are identified at an advanced stage, causing a five-year survival rate of less than 50% [2]. At present, the standard cancer treatment is mainly platinum chemotherapy for eliminating rapidly proliferating cells, but about 70% of patients relapse due to chemotherapy resistance [3, 4]. The complex regulatory network of tumor microenvironment (TME) has gradually become the focus of research, in which factors such as immune cell infiltration, metabolic reprogramming, and cell-cell interaction significantly affect tumor progression and treatment response [5]. In recent years, the lack of early diagnostic markers, the unclear mechanism of tumor microenvironment regulation, and the absence of a druggable driver oncogene for individualized treatment strategies are still the core scientific problems. Existing prognostic models usually rely on single omics data such as transcriptome or genomics, which makes it difficult to fully resolve the heterogeneity of TME [6]. Therefore, integrating multidimensional data to build a comprehensive prognostic model has become the key to improving the accurate diagnosis and treatment of OV. It is of clinical significance to explore the oncogene to improve the early-stage diagnostic rate of OV and provide a theoretical basis for breaking through the bottleneck of clinical treatment.

As a malignant tumor in the female reproductive system, the occurrence and development of OV are closely related to the complicated molecular mechanism of altered cellular metabolism and mitochondrial dysfunctions [7]. In the abdominal cavity, OV tumor emerges, metastasizes, and recurs, forming a unique microenvironment characterized by ascites, low oxygen, and low glucose. These conditions prompt tumor cells to switch to mitochondrial respiration for their survival [8]. Mitochondria is the core hub of cell energy metabolism and apoptosis regulation, which may be involved in tumor evolution through multiple pathways of metabolic recombination, reactive oxygen species (ROS) imbalance, and epigenetic regulation [9]. Scanning electron microscope examination of OV tissues shows an elevated count of mitochondria as well as cristae remodeling [10], potentially impacting mitochondrial bioenergetics. The increased mutation burden of mitochondrial DNA (mtDNA) is significantly associated with OV cases [11] suggesting the deregulation of the oxidative phosphorylation (OXPHOS) system. Mitochondrial genetic alterations are a significant hallmark of ovarian cancer, particularly affecting the OXPHOS pathway [12]. Abnormal expression of genes related to mitochondrial metabolism may affect the survival and metastasis of tumor cells through the regulation of ROS levels [13]. Since mitochondrial dysfunction poses a risk for OV tumorigenesis, discovering effective mitochondrial-related biomarkers for prognosis would be a promising area of study. However, there is still a lack of systematic analysis of the dynamic regulatory network and microenvironment interaction mechanism of Mitochondria-related genes (MRGs) in OV, and the clinical transformation of existing prognostic models is limited by tumor heterogeneity and insufficient standardization of detection.

Through integrating transcriptome and clinical data, this study aims to elucidate the multi-dimensional regulatory role of key MRGs in the occurrence and development of ovarian cancer and construct a novel prognostic evaluation system based on MRGs to provide a theoretical basis for precise treatment strategies.

## Material and methods

### Transcriptome data sources and manipulation

The RNA expression profile of ovarian cancer in the TCGA database and corresponding clinical data (n = 378) were selected to construct the model as the training group. The OV_AU dataset (n = 93) from ICGC database and ovarian cancer chip data of GSE17260 (n = 110), GSE26193 (n = 107), GSE26712 (n = 185), GSE30161 (n = 58), GSE49997 (n = 194), GSE63885 (n = 75), GSE9891 (n = 278), and GSE140082 (n = 380) were screened by the criteria of large sample size from the GEO database, serving as the validation groups. All data was converted into TPM format through a log2 conversion. We downloaded five pan-cancer immunotherapy datasets, containing Melanoma_GSE91061 (n = 109), Melanoma_GSE93157 (n = 25), Melanoma_Nathanson_2017 (n = 24), Melanoma_PRJEB23709 (n = 91), and IMvigor210 (n = 348).

GSE26712 (Normal = 10, Tumor = 185) and GTEx (Normal = 88) datasets were performed to screen differentially expressed genes (DEGs) between tumor and normal ovary tissues. Data correction of the chip data was conducted by the normalizeBetweenArrays function in the limma package. The elimination of batch effects between data was performed via the Combat function in the sva R package. Principal component analysis (PCA) was performed to evaluate batch effects between datasets. The copy number variation (CNV) of each patient was analyzed using GISTIC 2.0 software and the tumor mutational burden (TMB) of each patient was calculated using maftools R package.

### Processing single-cell RNA sequencing (scRNA-seq) data

The scRNA-seq datasets GSE235931(OV samples n = 15) and GSE184880(OV samples n = 7) were obtained for single-cell analysis by Seurat R package (version 4.1.3). Following cell quality control, the mitochondrial gene proportion was restricted to less than 20%, and hematopoietic cell contamination was maintained below 3%. For dataset GSE235931, cellular UMI and gene count thresholds were established as 200–15,000 and 200–5,000, respectively. In contrast, the corresponding ranges for GSE184880 were set at 200–50,000 for UMI counts and 200–10,000 for gene counts.

Data normalization was conducted by the function of NormalizeData. We selected 2,000 highly variable genes through the function of FindVariableFeatures. To remove cell cycle effects, data transformation was carried out using the argument vars.to.regress = c(" s.core ", "G2M.Score" from the function of ScaleData from the Seurat package. Batch effects were processed via harmony. Dimensional-reduction method and clustering algorithm were from Seurat. We performed the FindAllMarkers function to calculate differential genes between clusters or cell types with the threshold of p < 0.05, log2FC > 0.25, and a ratio of expression > 0.1.

### Establishment of prognostic mitochondria signature (MS) model

We screened mitochondria-related DEGs by limma R software at the threshold of |logFC|> 1 and p < 0.05. Univariate Cox analysis was performed to assess the prognostic genes for OV patients (p < 0.05). The construction of the prognostic MS model was based on 101 combinations of 10 machine learning algorithms at a leave-one cross-validation framework. The comprehensive algorithms include Random survival Forest (RSF), Elastic Network (Enet), Lasso, Ridge, Stepwise Cox, CoxBoost, Cox Partial least squares Regression (plsRcox), Supervised Principal Component (SuperPC), Generalized enhanced regression model (GBM) and survival support vector machine (Survival-SVM).

The whole signature generation procedure is as follows: (a) Univariate Cox regression analysis identifies prognostic genes in the combined data set including TCGA (described in the previous step); (b) Then, 101 algorithm-based combinations of prognostic genes were performed to fit a prediction model based on leave-one cross-validation framework in the TCGA-OV cohort; (c) All models are detected across the validation groups; (d) For each model, the Harrell Consistency Index (*C-index*) was calculated across datasets. We then ranked the predictive performance of the models based on the mean *C index*. The model with the highest average *C-index* was considered the best, showing both robust performance and clinically translational significance. According to the cutoff value of the group determined by the surv_cutpoint function, OV patients were divided into high-MS and low-MS groups. The “timeROC” package was performed to conduct ROC analysis to evaluate the sensitivity and specificity of the MS in predicting OS at 2, 3, and 5 years in OV patients. Furthermore, the accuracy of MS was evaluated using PCA. A total of 32 published prognostic signatures from published literature, including lncRNA and mRNA, were compared with the MS model to assess the prognostic performance, with *C-index* acting as the evaluation metric.

### Analysis of cell–cell communication

Potential differences in cell-to-cell communication modules were evaluated using CellChat package. The normalized gene expression matrix was imported using the CellChat function to create CellChat objects. Then we performed the identifyOSAerExpressedGenes, identifyOSAerExpressedInteraction, and ProjectData function using the default parameters for preprocessing of the data. The computeCommunProb, filterCommunication, and computeCommunProbPathway functions were applied to identify any potential ligand-receptor interactions. Finally, the aggregateNet function was conducted to generate the cell communication network.

### Analysis of immune infiltration

The abundance of immune cells within tumor samples in each patient from the TCGA cohort was determined using IOBR software, which contained seven algorithms to assess immune infiltration. These data were applied to plot heat maps and quantify the relative proportion of immune infiltration in TME.

### Drug sensitivity and predicting response to immunotherapy

We performed the oncoPredict R package to calculate semi-maximum inhibitory concentration (IC50) and AUC values for common chemotherapy drugs based on GSDCv2 and CTRP (https://portals.broadinstitute.org/ctrp.v2.1/) databases to assess the relationship between risk scores and drug sensitivity. The Wilcoxon rank sum test was performed to compare IC50 values or AUC values between risk groups.

The Tumor Immune Dysfunction and Exclusion (TIDE) algorithm (https://tide.nki.nl) was performed to predict immune responses in the TCGA dataset to evaluate MS differences in immunotherapy response. The Cancer Immunome Atlas (https://tcia.at/home) assesses the immunophenoscore (IPS) of OV patients and identifies populations suitable for immunotherapy.

### Cell lines

The A2780 and SKOV3 human ovarian cancer cell lines and IOSE-80 human normal ovarian epithelial cells were acquired by Sigma-Aldrich (St Louis, Missouri, USA). These cells were grown at 37 °C in an incubator with 5% CO_2_ in RPMI-1640 medium (Sigma-Aldrich, St Louis, Missouri, USA), supplemented with 10% FBS, 2 mM glutamine, 100 U/mL penicillin, and 10 mg/mL streptomycin (Gibco, ThermoFisher Scientific, Waltham, MA, USA). Cells were regularly evaluated for Mycoplasma contamination.

### RNA extraction and qRT-PCR assays

RNAs in IOSE-80, A2780, and SKOV3 cell lines were extracted with RNAiso Plus (Takara, Tokyo, Japan) and were reversely transcribed into cDNA using PrimeScriptTM RT Master Mix (Takara). Quantitative real-time PCR (qRT-PCR) was performed by TB Green ®Premix Ex TaqTM II (Takara). β-actin was used as reference genes.

### Statistical analysis

All analyzing data are carried out in R software (v.4.1.3). The correlation between two continuous variables was evaluated by the Pearson correlation coefficient. Categorical variables were compared by Chi-square test and continuous variables were compared by Wilcoxon rank sum test or t test. The survminer package was performed to determine the best cutoff value. Cox regression and Kaplan–Meier analysis were performed using a survival package.

## Results

### Identification of mitochondria-related prognostic DEGs for OV

**Fig. 1:**
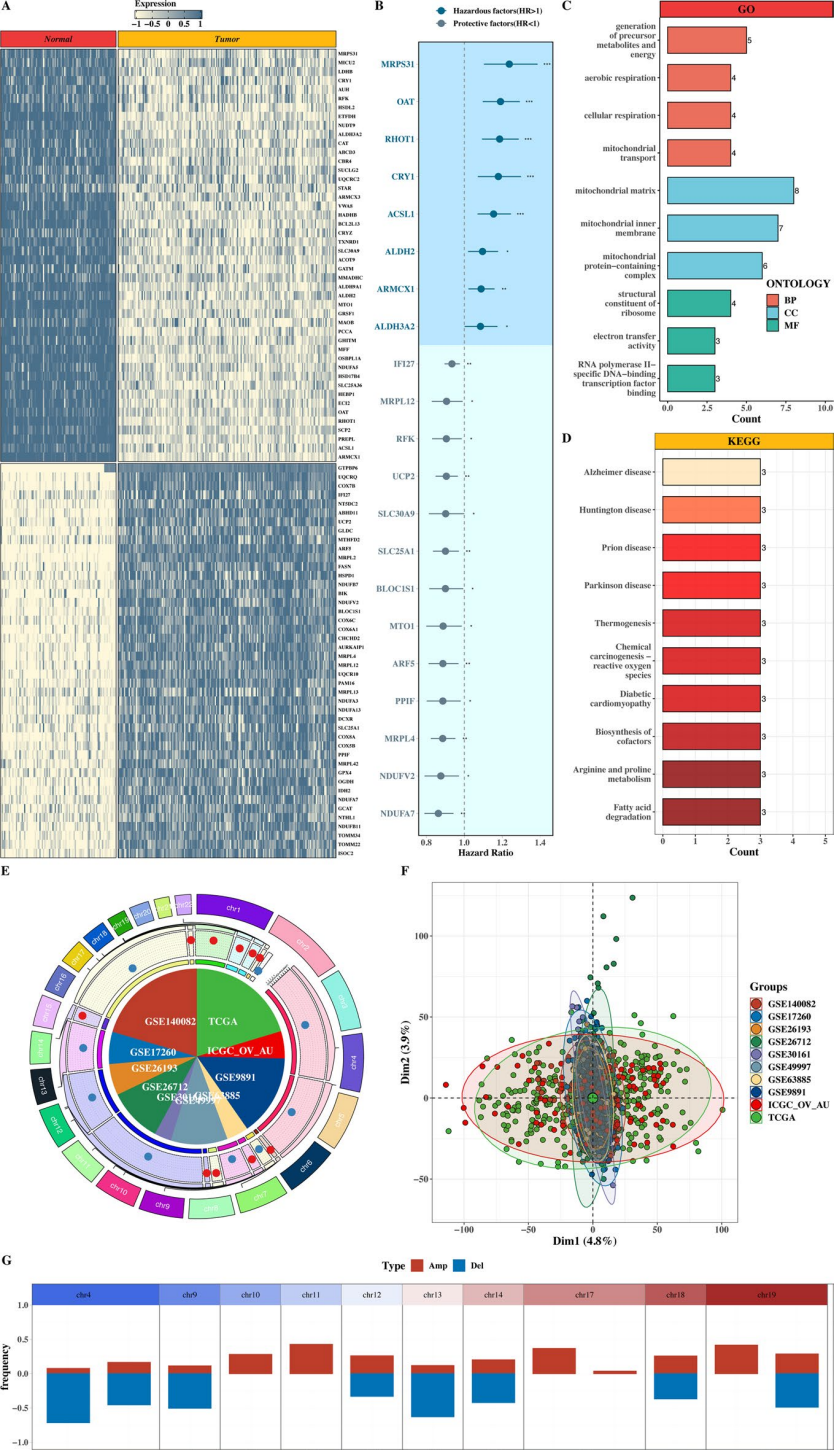
Identification of mitochondria-related prognostic DEGs for OV. **A** Heat map of mitochondria-related DEGs. **B**. Forest map for 21 significant prognostic genes by univariate Cox analysis. **C**-**D** GO and KEGG enrichment histogram. **E** Circos map of the distribution of mitochondria-related prognostic genes in the human chromosome. **F** PCA plots of each OV dataset for batch effect removal. **G**. CNV plot of prognostic genes within chromosomal locations.

We obtained mitochondria-related DEGs from OV tissues and normal ovary tissues from GSE26712 and GTEx datasets (Fig. 1A). Both GSE26712 and GTEx datasets were carried out into a PCA analysis for removing batch effect (Suppl Fig. 1A). 21 prognostic genes were screened by univariate Cox analysis and divided into 8 hazardous factors and 13 protective factors with significance according to the Hazard Ratio (Fig. 1B). Their expression profiles were visualized in Suppl Fig. 1B. Subsequently, GO and KEGG enrichment analysis was performed on this prognostic gene set, demonstrating that these genes were enriched in mitochondrial matrix, mitochondrial inner membrane, and mitochondrial protein-containing complex and most of them functioned in the biological process (BP) such as generation of precursor metabolites and energy (Fig. 1C-D). Figure 1E visualized the location of each gene in the human chromosome. PCA plot undergoing batch effect removal was exhibited for each OV transcriptome dataset to render certain comparability between datasets (Fig. 1F). The CNV plot of each gene located in diverse chromosome was introduced in Fig. 1G, suggesting frequent changes in mitochondria-related prognostic genes.

### Construction and validation of MS model

**Fig. 2:**
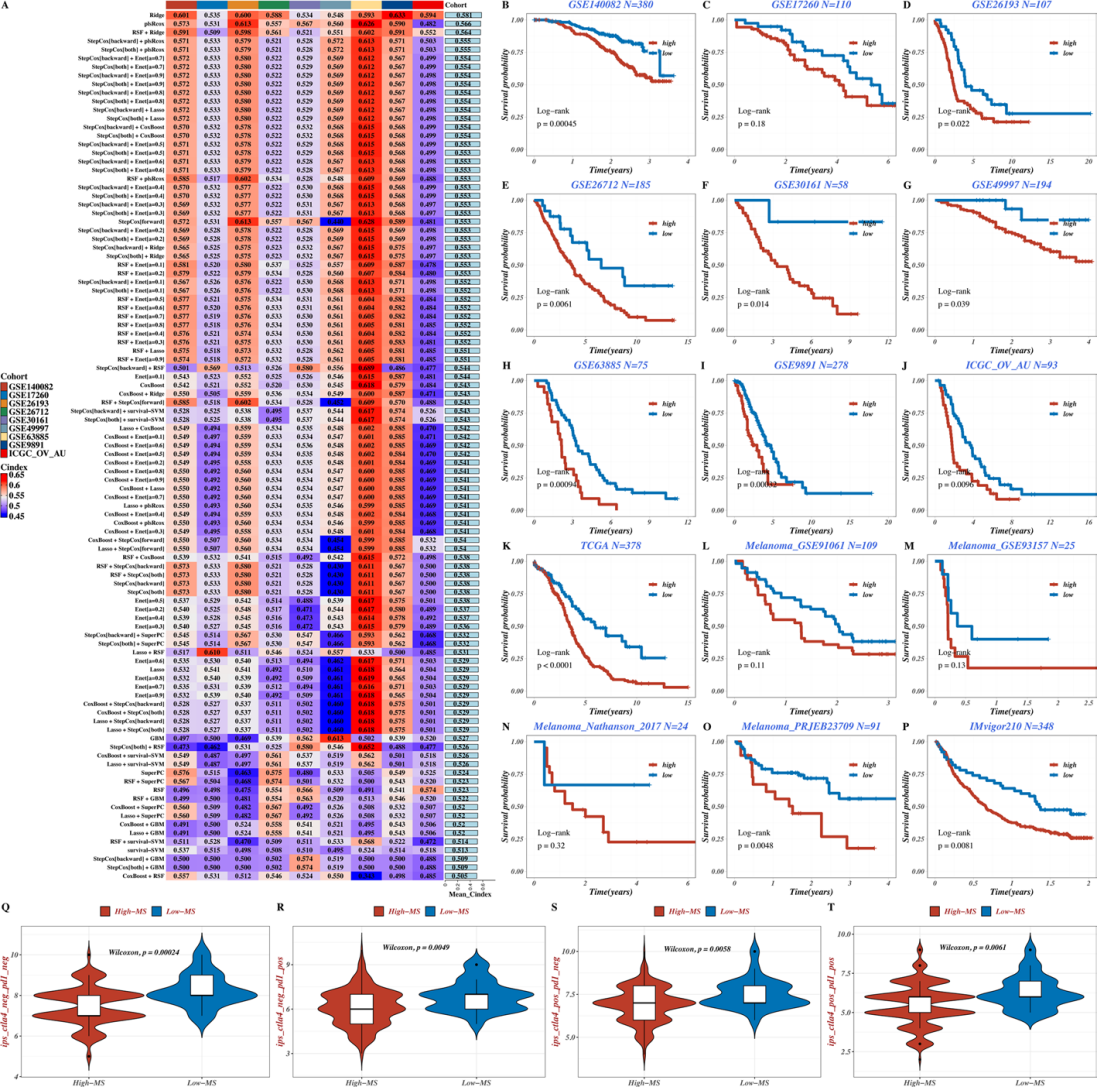
Construction and validation of MS model. **A** Heat map of C index of 101 combination algorithms in nine validation datasets. **B**-**K** Survival analysis results of training and validation datasets of OV. **L-P**. Survival analysis results of pan-cancer immunotherapy datasets. **Q**-**T** Violin chart of IPS differences between MS groups showing the sensitivity to immunotherapy.

The TCGA dataset acted as the training cohort, while eight GEO datasets and ICGC_OV_AU (n = 93) dataset were employed for validation. The criterion for selecting the model was the average *C-index* from the nine validation cohorts. Therefore, we acquired the expression profiles of mitochondria-related prognostic DEGs from nine validation datasets based on 101 combinations of 10 machine learning algorithms, showing that Ridge was selected as the optimal model (Fig. 2A). The K-M curves of training and validation datasets were plotted to identify the survival results, suggesting that patients in high-MS group have the significantly inferior OS than that in low-MS group (p < 0.05 except for GSE17260, Fig. 2B-K). Additionally, five pan-cancer immunotherapy datasets (including 4 melanoma cohorts and IMvigor 210 cohort) were applied to predict its survival results through MS scores, suggesting higher suitability of low-MS group for immunotherapy with a better prognosis (Fig. 2L-P). IPS values from the TCIA website were utilized to evaluate significant differences in immunotherapy between MS groups, indicating that low-MS group is more appropriate for treatment with PD-1, CTLA-4, or their combination therapy (all p < 0.01, Fig. 2Q-T). The functional enrichment analysis of MS was performed in Suppl Fig. 2.

### Prognostic performance of MS model

**Fig. 3:**
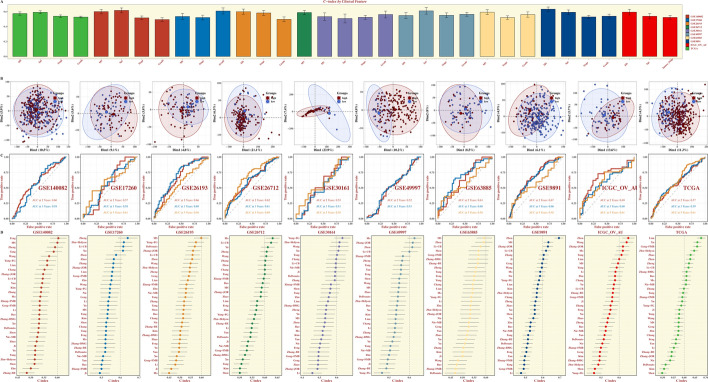
Prognostic performance of MS model. **A** Bar chart comparing *C-index* of age, Stage and Grade in each dataset. **B**-**C** PCA plots and timeROC results of each OV dataset. **D** Comparison results of *C-index* values between MS model and other models reported in 32 literatures in each OV dataset.

We compared the *C-index* values of clinical features such as age, stage, and grade in each OV dataset (Fig. 3A). The PCA analysis results showed that the expression levels of the model genes could distinguish OV patients in high and low MS groups (Fig. 3B). The timeROC curves further identified that MS score could reliably predict the prognosis of OV patients across each dataset (with 2-, 3-, and 5-year AUC values generally more than 0.55, Fig. 3C). In addition, the *C-index* value of MS model was compared with that reported in 32 other literatures across 10 datasets, revealing the excellent prognostic value of MS model for OV in most datasets (Fig. 3D). The CNV results were listed in Suppl Fig. 3.

### Immune infiltration analysis

**Fig. 4:**
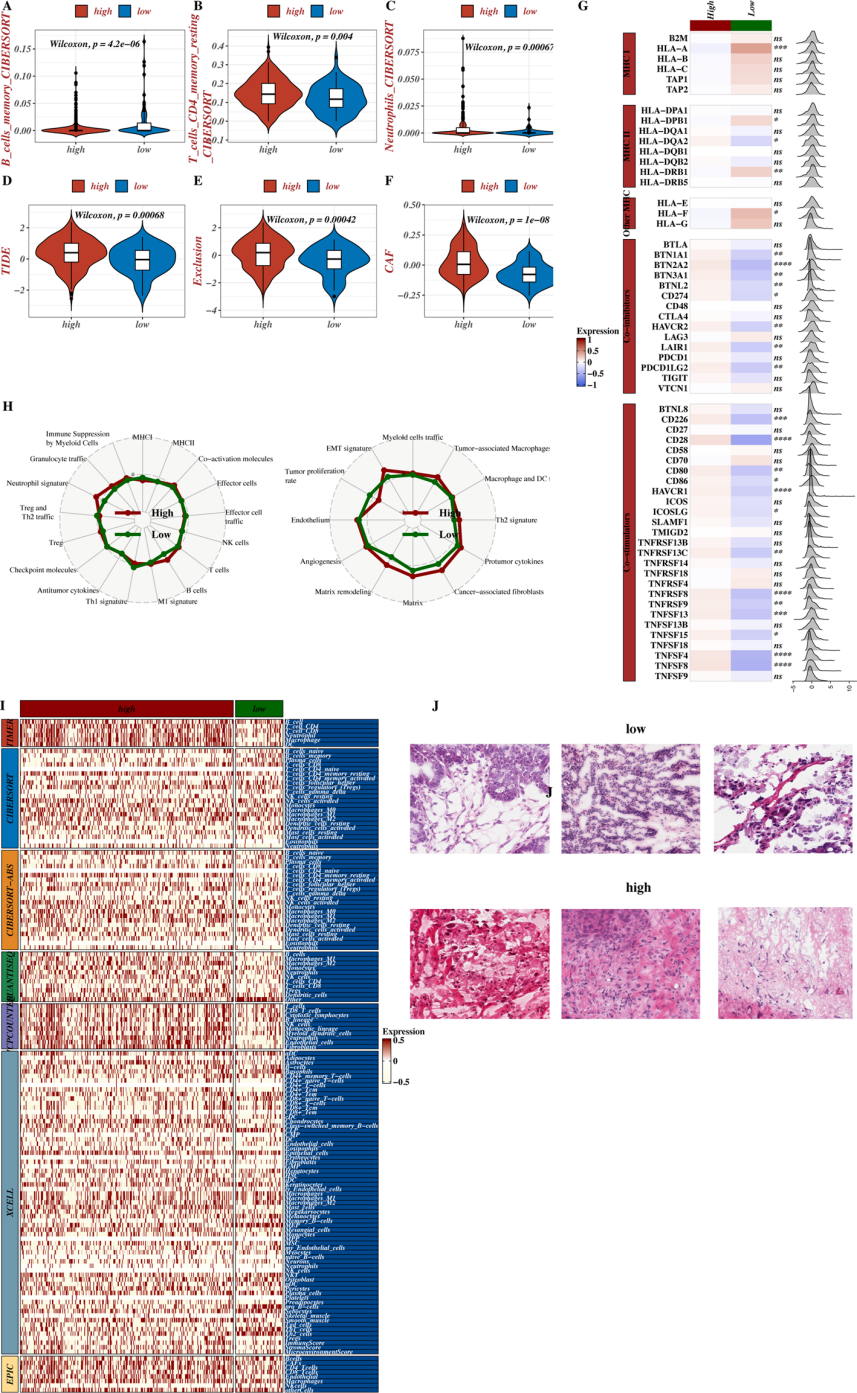
Immune infiltration results. **A**-**F** CIBERSORT and TIDE algorithms predicted the difference of TME in high and low MS groups. **G** Heat map of expression of immune regulatory genes in high and low MS groups. **H** Radar map of differences of immune-related enrichment pathways in high and low MS groups. **I**. Heat maps of immune infiltration differences evaluated by seven software between groups. **J**. Identification of pathological sections of high and low MS group samples from TCGA database.

The CIBERSORT and TIDE algorithms were conducted to predict TME and the significant difference of CD4 + T cells, B cells, neutrophils, and cancer-associated fibroblasts (CAFs) between the high and low MS group (all p < 0.01, Fig. 4A-F). We exhibited the expression of immune regulatory genes in both groups, indicating that the low MS group appeared to activate MHC class I molecules and suppress most co-inhibitory molecules and co-stimulatory molecules (Fig. 4G). Furthermore, we performed the ssGSEA algorithm to calculate the abundance of immune infiltrating cells and immune-related pathway activity between the high and low MS groups, showing the high MS group had higher cell abundance of Tregs, B cells, T cells, M1 signature, neutrophil signature, tumor-associated macrophages, and the activation of matrix remodeling, angiogenesis, EMT-related immune pathways (Fig. 4H). The results of immune infiltration evaluated by seven software were shown between the high and low groups (Fig. 4I). To confirm the immune infiltration results, we acquired H&E pathological maps from the TCGA database for the high and low MS groups, illustrating more lymphocyte infiltration in the high MS group in Fig. 4J.

### MS distribution and cellular interactions based on single cell data

**Fig. 5:**
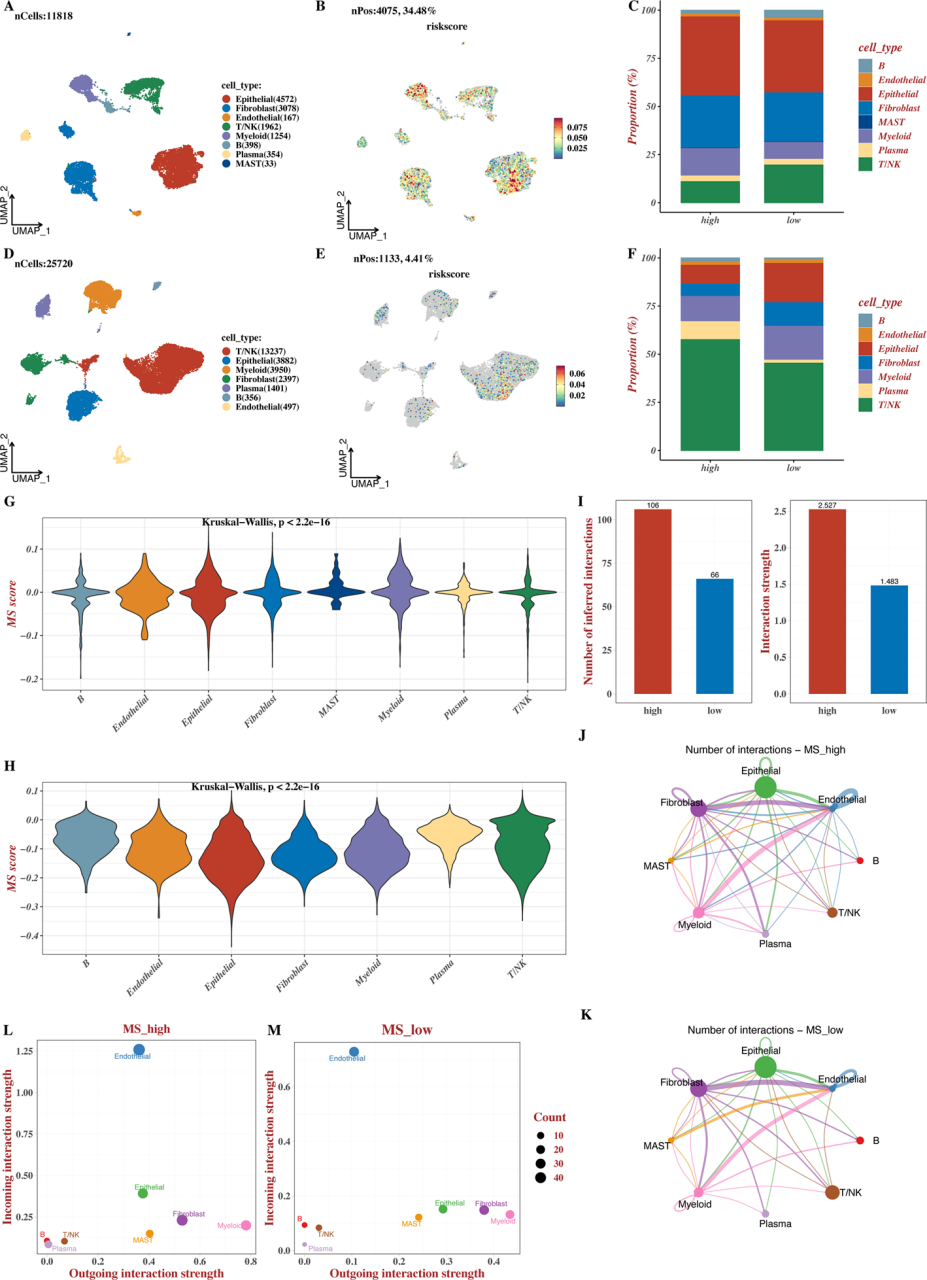
MS distribution and cellular interactions based on single cell data. **A**-**C** Single-cell analysis result of GSE235931. **D**-**F** Single-cell analysis result of GSE184880. **G**-**H** Violin plot of MS scores of each cell subtype by the GSE235931 and GSE184880 datasets. **I**-**M** Results of cell-cell interactions and communication differences between high and low MS groups

Based on single-cell data of GSE235931 and GSE184880, cell annotation results were revealed in Fig. 5A, D and the distribution of MS was visualized in Fig. 5B, E. The proportion of diverse annotated cell types was calculated between high and low MS groups across two datasets (Fig. 5C, 5F). Violin plots indicated that NK/T cells had the highest MS scores (p < 2.2e-16, Fig. 5G, H), indicating that higher MS scores may be associated with greater immune cell infiltration potential. Furthermore, we determined cellular interactions between high and low MS groups (Fig. 5I–K) and found stronger cell-cell communication and more intense outgoing and incoming signals in the high MS group using CellChat software (Fig. 5L–M). Different communication between high and low MS groups was evaluated in Suppl Fig. 4 based on single cell data.

### Validation of ornithine aminotransferase (OAT) gene

**Fig. 6:**
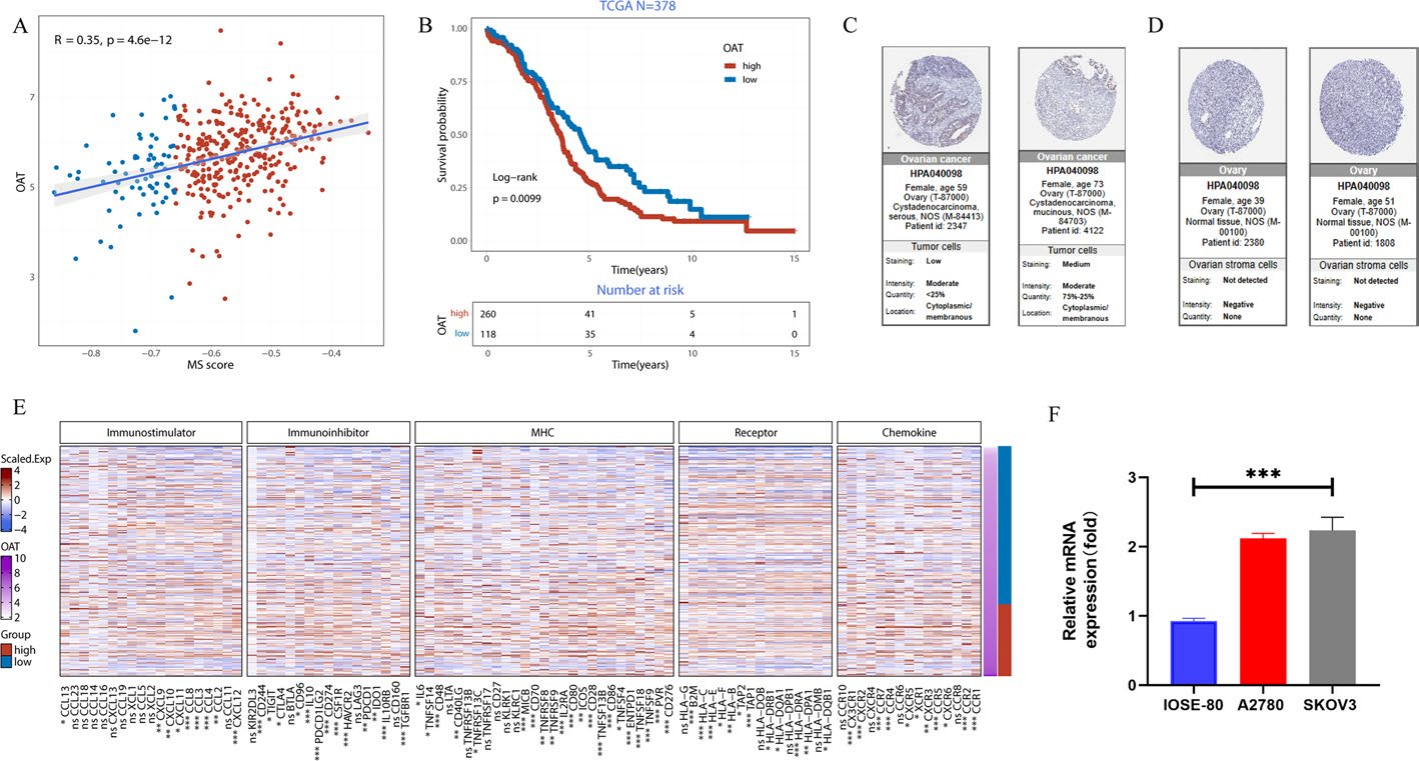
Validation of OAT gene. **A** Correlation of MS score and OAT. **B** Survival analysis of patients with high or low OAT expression. **C**-**D** Immunohistochemical results of OAT in tumor and normal ovary tissues from HPA database. **E** Heat map of correlation between OAT and immune-related genes. **F** The mRNA expression of OAT in OV cells. ***p < 0.001

Previous analyses have demonstrated that OAT is a risk gene (HR > 1, p < 0.05, suppl 1B). Figure 6A revealed a significant positive correlation between OAT and MS score (correlation = 0.35, p < 0.05). K-M curves showed that OV patients with highly expressed OAT had a worse prognosis than those with lowly expressed OAT (p < 0.01, Fig. 6B). It was reported in the HPA database that OAT was detected in OV tissue with no existence in normal ovary tissue based on immunohistochemistry (Fig. 6C-D). Heat map of the correlation between OAT and immune-related genes was shown in Fig. 6E, suggesting a potential role for NME4 in regulating tumor immunity. Following the qRT-PCR assay, overexpressed OAT mRNA was determined in A2780 and SKOV3 human ovarian cancer cell lines compared to the normal IOSE-80 ovary cells (p < 0.05, Fig. 6F). The other proteins and drug correlations with MS were visualized in Suppl Fig. 5. IC50 values will be correlated with gene expression signatures to validate oncoPredict’s accuracy. Further drug sensitivity tests (MTT or CellTiter-Glo assays) should be performed on SKOV3 or A2780 cell lines treated with predicted drugs.

## Discussion

OV is a gynecological malignancy with high molecular heterogeneity. Chemotherapy-resistant mechanisms seriously restrict the clinical prognosis of OV. In recent years, mitochondrial energy metabolic reprogramming has been proven to be a key feature of malignant tumors [14] due to the alteration of mitochondrial energy metabolism adapting to high energy demands of growth and movement of tumor cells [15]. Overproduction of mitochondrial ROS (mtROS) promotes apoptosis and metastasis in cancer cells, due to its capability of triggering the apoptotic pathway [16]. It is reported that ROS-mediated reduction in the internalization of tumor-derived exosomal miR-155 by macrophages promotes macrophage infiltration and induces T cell dysfunction in ovarian cancer, characterized by upregulated programmed death ligand 1 (PD-L1) [17]. Mitochondrial metabolite-mediated immune suppression was widely studied, supporting the importance of the potential mechanism of mitochondrial metabolism and immune escape. In this study, researchers comprehensively investigated the prognostic and immunological implications of mitochondrial signature (MS) across transcriptome, clinical data and single-cell sequencing of multiple OV cohorts. 21 DEGs related to mitochondrial function and immune regulation were screened by omics data. Model gene OAT is not only significantly correlated with the overall survival of patients, but also deeply involved in the regulation of tumor metabolic microenvironment. High expression of OAT may promote the infiltration of M2 macrophages, which highly express immunosuppressive molecules such as PD-L1 [18]. Our findings provide a new perspective for elucidating the progression mechanism of OV.

First, we identified a panel of mitochondrial-associated DEGs with significant prognostic value. Hazard ratio analysis distinguished hazardous (hazard ratio, HR > 1) and protective (HR < 1) factors, implicating dysregulated mitochondrial electron transport and ribosomal activity in tumor progression. GO and KEGG analyses further highlighted the enrichment of mitochondrial-associated genes in pathways of generation of precursor metabolites and energy, consistent with prior studies linking metabolic reprogramming to cancer aggressiveness [19]. Chromosomal distribution analysis revealed frequent alterations in chr4, chr9, and chr17, regions previously associated with cancer-related genomic instability [20]. The prognostic MS model was constructed based on 101 combinations of 10 machine learning algorithms, with Ridge achieving the highest mean *C-index* (0.581). Survival curves confirmed significant stratification between high- and low-MS groups, underscoring its clinical applicability in the prognosis of MS. Notably, the model demonstrated superior predictive performance in traditional clinical parameters such as age, stage, and grade in predicting outcomes, emphasizing the added value of mitochondrial gene signatures. Following the comparisons with models from other publications, we ranked the first to construct a mitochondrial-related prognostic model, revealing the excellent prognostic value. Additionally, immune landscape analysis revealed pronounced differences between MS groups. High-MS group exhibited elevated neutrophil infiltration (p = 0.00067), cancer-associated fibroblasts (CAFs) activity (p = 1e-08), memory B cells (p = 4.2e-06), and CD4 + T cells (p = 0.004), suggesting its potential resistance to immunotherapy. Single-cell analysis showed that MS scores were significantly elevated in the high-MS epithelial cells and interaction with fibroblasts was enhanced, suggesting that metabolism-interstitial cross dialogue may drive immunosuppression. High-MS group showed significantly upregulated immune checkpoints (e.g., PDCD1LG2) compared to low-MS group, suggesting potential responsiveness to checkpoint inhibitors despite immunosuppressive tendencies. Cell-cell interaction analysis revealed stronger communication networks in high-MS group, driven by pro-inflammatory signaling between fibroblasts and immune cells. This echoes recent work emphasizing stromal-immune crosstalk in metabolic adaptation [21].

OAT gene has been pinpointed as a potential biomarker and a target for therapy in human cancers such as gastric cancer [22], hepatocellular carcinoma [23], and non-small cell lung cancer [24]. It serves as a β-catenin target gene, the overexpression of which is associated with activation of β-catenin to regulate glutamine metabolism. Once activated, β-catenin signaling drives the expression of target genes that are critical for cell cycle progression and contributes to the initiation of the regeneration process [25]. Among the signaling cascades that are deregulated in cancer, the Wnt/β-catenin signaling pathway plays a role in oncogenesis, while inhibition of β-catenin signaling in tumor cell lines has an antitumor effect [26]. In addition, OAT-mediated oxidative stress may inhibit the activity of cytotoxic T cells by activating the TGF-β pathway to regulate immune escape mechanisms. In idiopathic pulmonary fibrosis models, overexpression of OAT could promote the production of mtROS by activating proline dehydrogenase, in turn activating TGF-β signals [27]. Ornithine consumption mediated by OAT may lead to enhanced glutamine decomposition, which generates excessive mtROS and may induce PD-L1 expression through AP-1 pathways [28]. In this study, we identified the overexpression of OAT gene in clinical OV tissues and in-vitro tumor cells, which is significantly positively correlated with MS score. It shows excellent prognostic value for OV, showing inferior overall survival in OV patients with highly-expressed OAT than the lowly-expressed groups. These findings suggested the potential of OAT as a novel biomarker for the diagnosis and therapeutic outcome monitoring of OV. A new therapeutic strategy for OV may be designated by targeting the inhibition of OAT. However, no molecules targeting any component of the Wnt/β-catenin pathway are currently being tested in clinical trials for the treatment of OV.

Limitations of the current study were indicated. First, the causal effects of key genes need to be clarified through CRISPR interference experiments. The further validation section should introduce the OAT protein level validation in independent external cohorts and clinical samples due to limited single-cell sample size and unintegrated proteomic data. In addition, this study did not fully consider the threshold-dependent net benefit analysis in clinical scenarios. It is a trend to add a decision curve analysis (DCA). Second, cross-platform testing standardization affects clinical applications. Future studies should integrate metabolomics data to build a multidimensional prediction system and verify the molecular mechanism of MRGs regulation of chemotherapy sensitivity in knockdown/rescue assays. Third, potential bias was introduced by using GTEx healthy ovarian tissues (non-matched to tumors) instead of adjacent non-tumor tissues from OV patients. DEG analysis should be supplemented with matched tumor-normal pairs. The establishment of this model not only provides a new tool for the stratified treatment of ovarian cancer but also lays a theoretical foundation for the development of therapeutic strategies targeting mitochondrial metabolism.

## Conclusion

This study establishes a mitochondrial gene-based signature as a robust prognostic tool and reveals its interplay with immune suppression. Targeting mitochondrial pathways or stromal-immune interactions may offer novel therapeutic avenues for OV patients.

## Supplementary Information

Supplementary material 1: Suppl Figure 1. A. PCA plots of TCGA and GTEx data before and after batch effect removal. B. Heat map of expression of 21 modeling genes.

Supplementary material 2: Suppl Figure 2. A-G. Functional analysis of Msigdb gene sets between high and low MS groups. H. Correlation analysis of MS model with TIP tumor immunity and immunotherapy pathways.

Supplementary material 3: Suppl Figure 3. A-B. CNV results obtained from the gistic2.0 software in high and low MS groups. C. The mutation results based on TCGA mutation data calculated by maftools software. D. The difference between high and low MS groups in Broad and Focal aspects of the CNV results. E. K-M curves of the TMB values combined with MS score in different groups.

Supplementary material 4: Suppl Figure 4. Heat maps of communication between high and low MS groups in single cell data.

Supplementary material 5: Suppl Figure 5. A-H. Correlation between MS and protein abundance. I-P. The relevance of MS to CERES. Q-T. Relevance of MS to drugs from GDSCv2 and CTRP databases.

## Data Availability

All data can be requested from the first author.
