# Supplementary figures and images for: Construction of mitochondrial signature (MS) for the prognosis of ovarian cancer
Source: Discov Oncol. 2025 Jul 22;16:1388. doi: 10.1007/s12672-025-02892-7 (PMC12283533; doi:10.1007/s12672-025-02892-7)

A

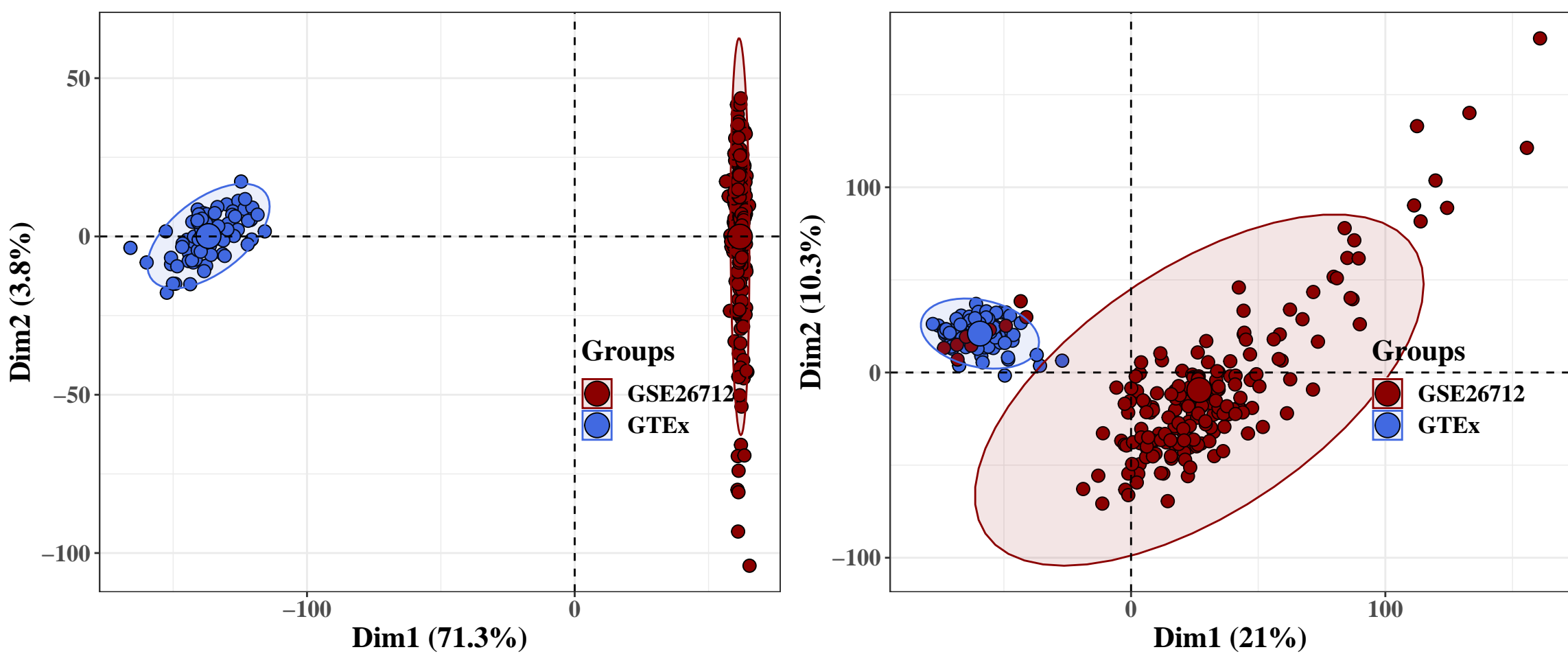

B

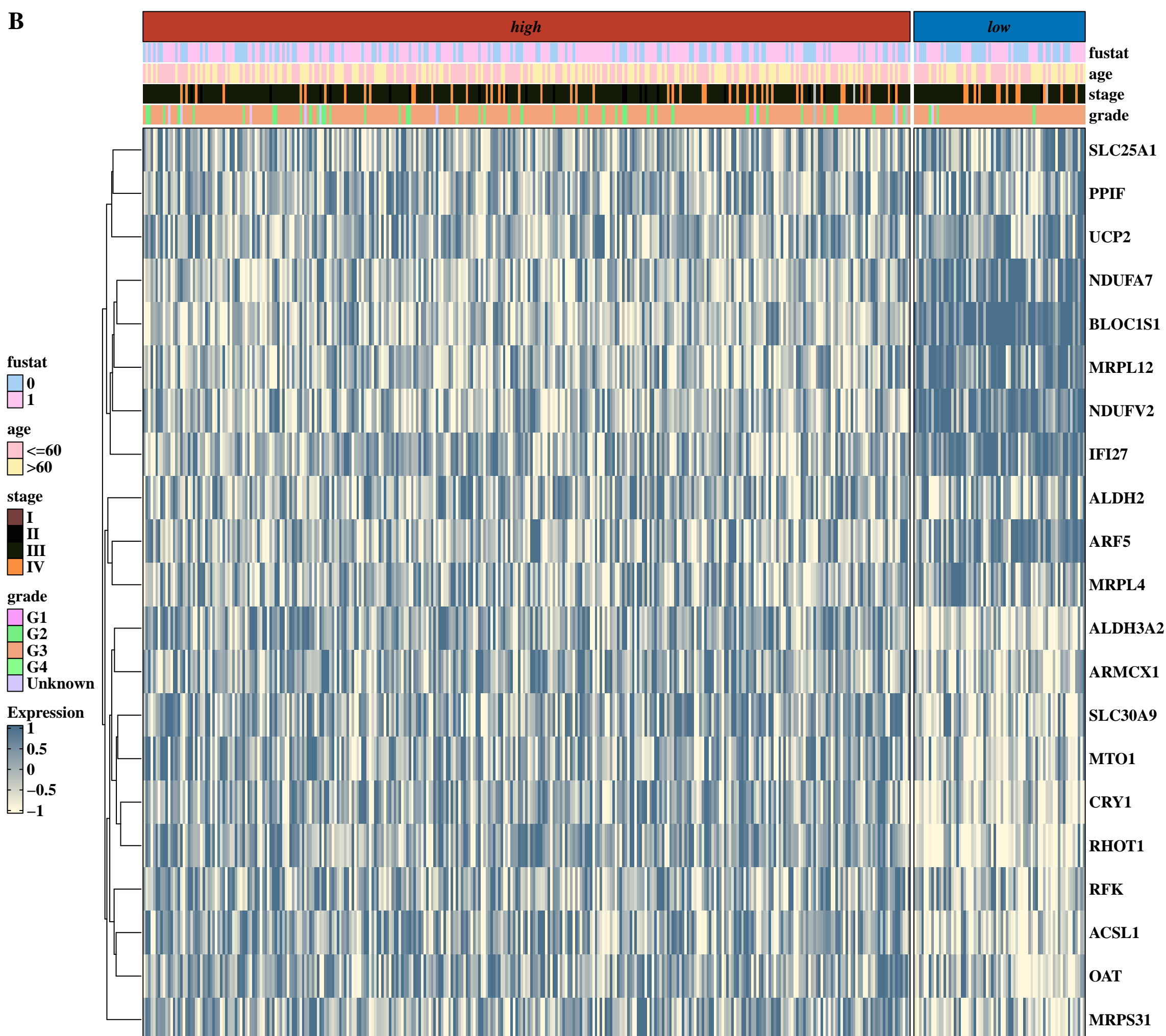

Supplement: Supplementary file 1 — Supplementary material 1: Suppl Figure 1. A. PCA plots of TCGA and GTEx data before and after batch effect removal. B. Heat map of expression of 21 modeling genes. [file 12672_2025_2892_MOESM1_ESM.pdf]

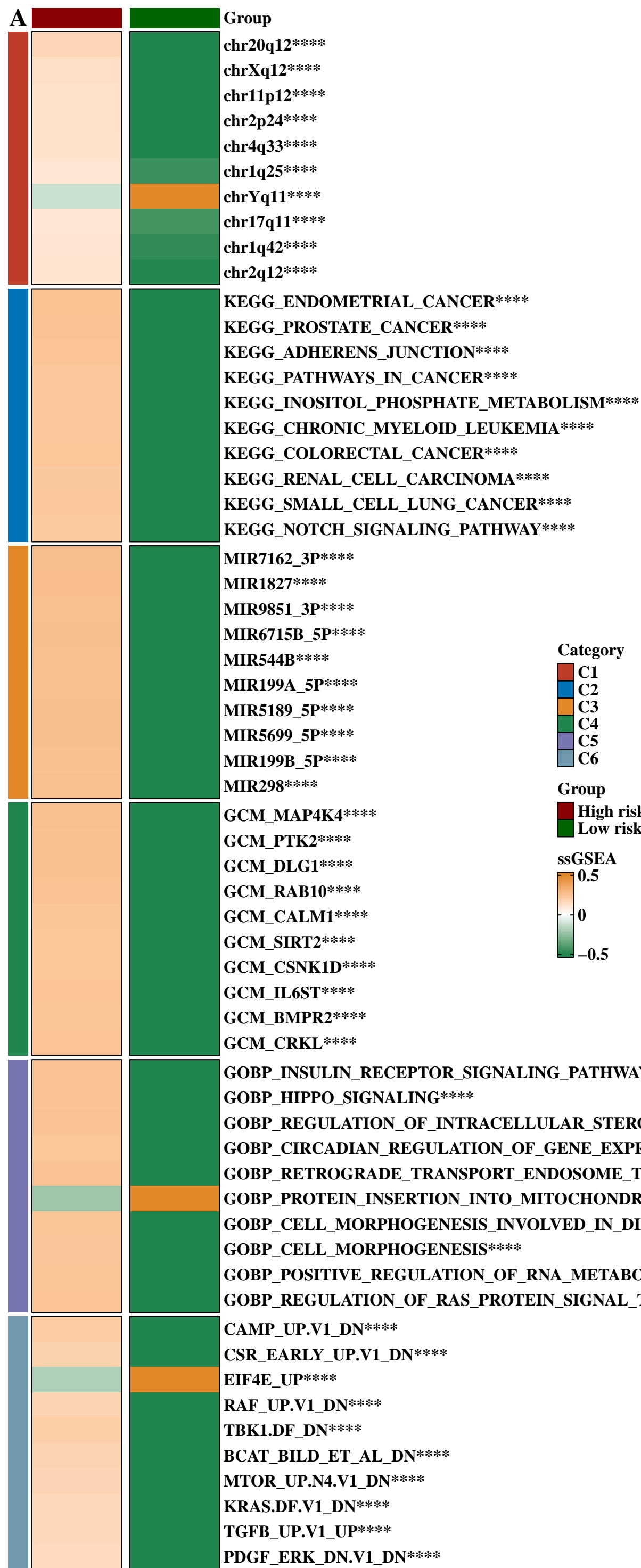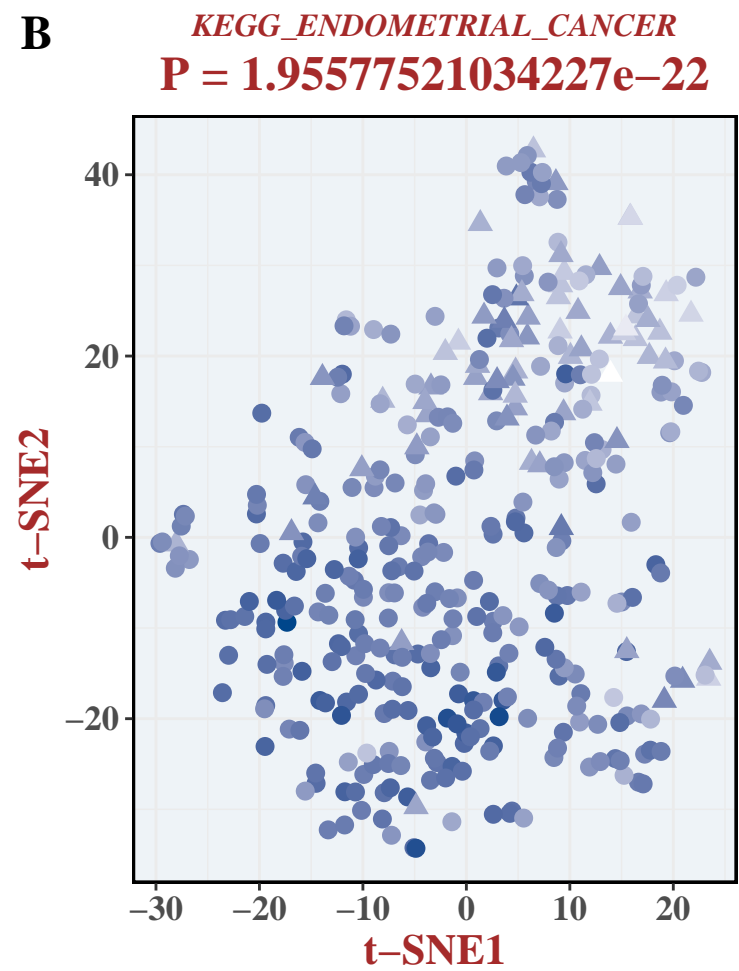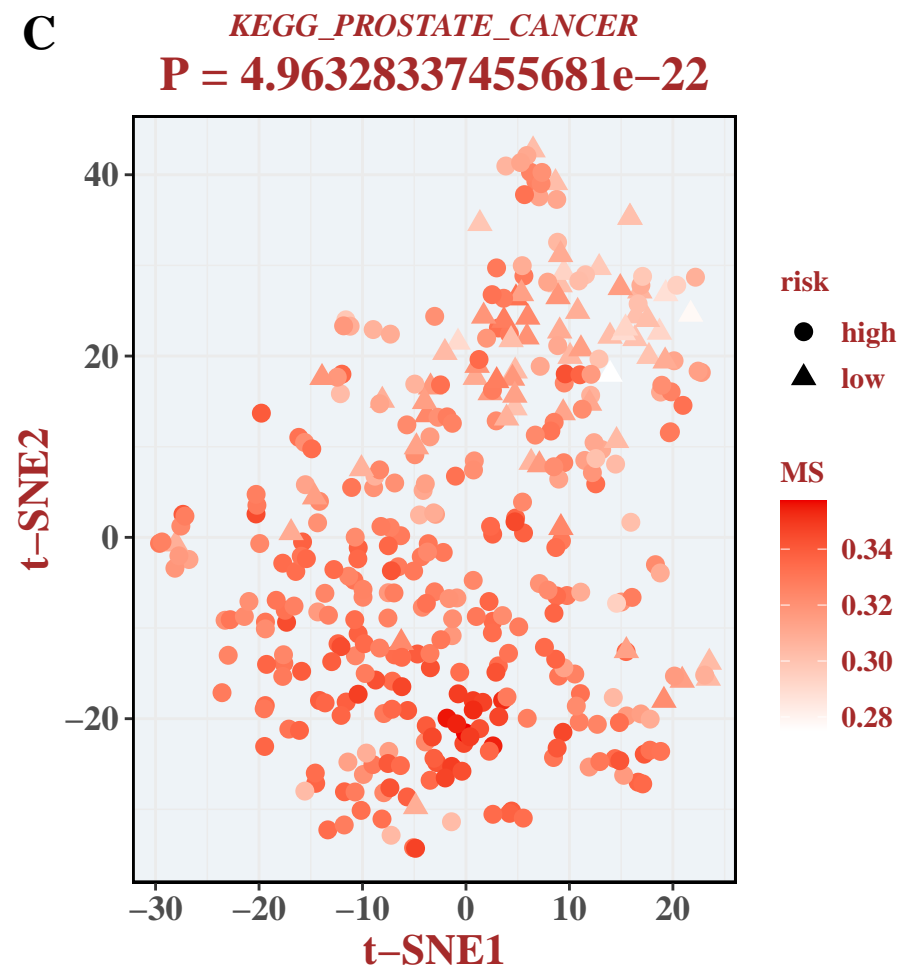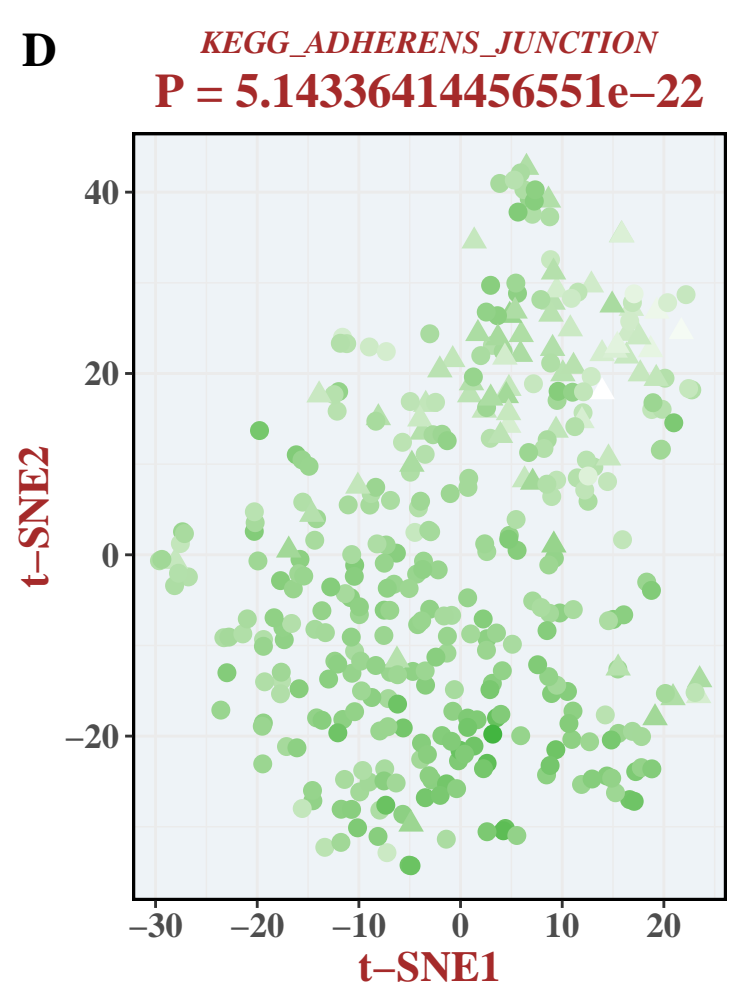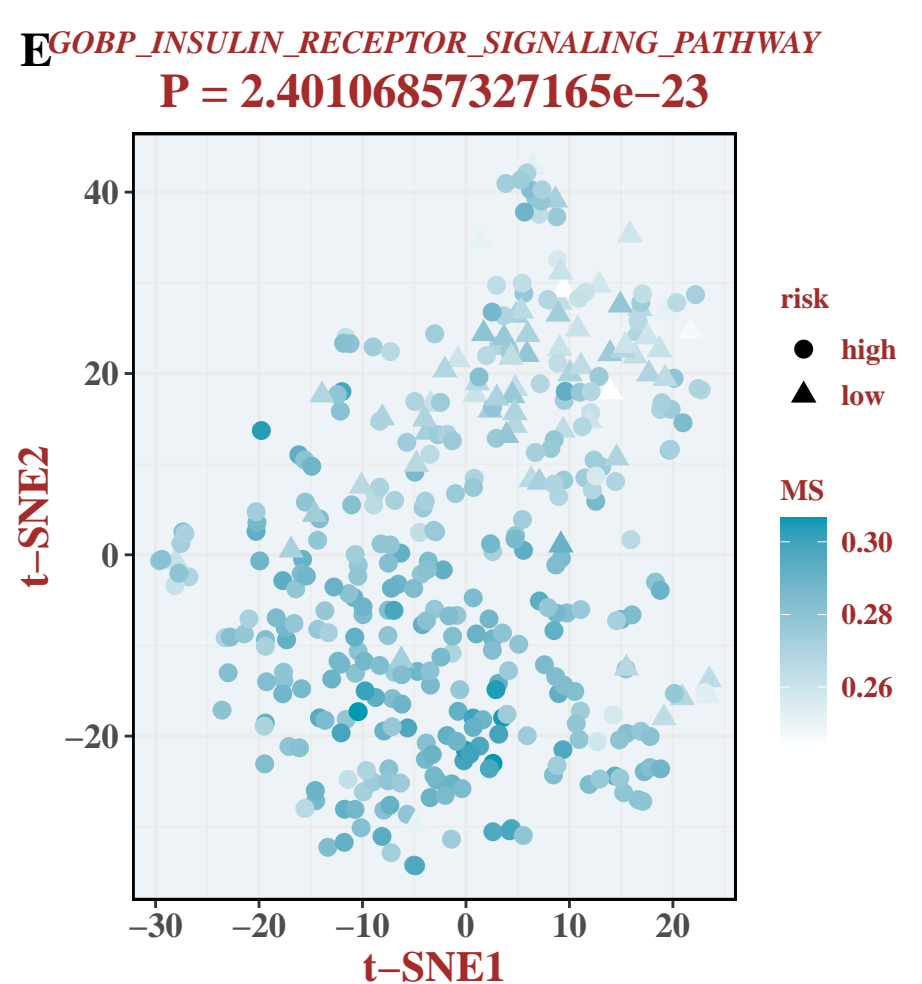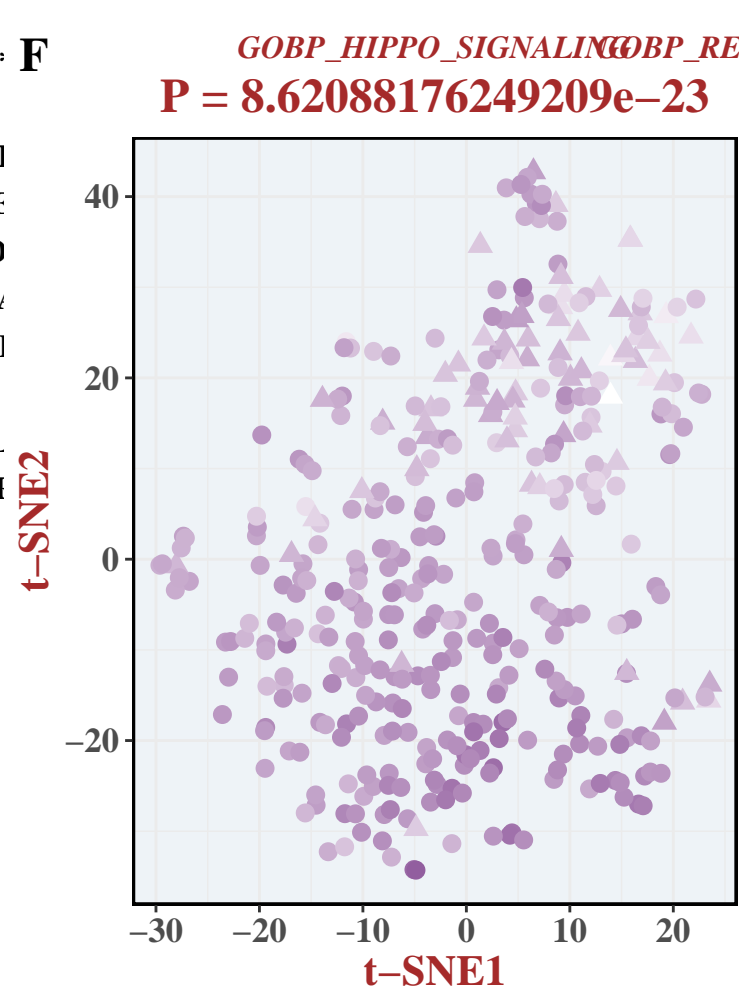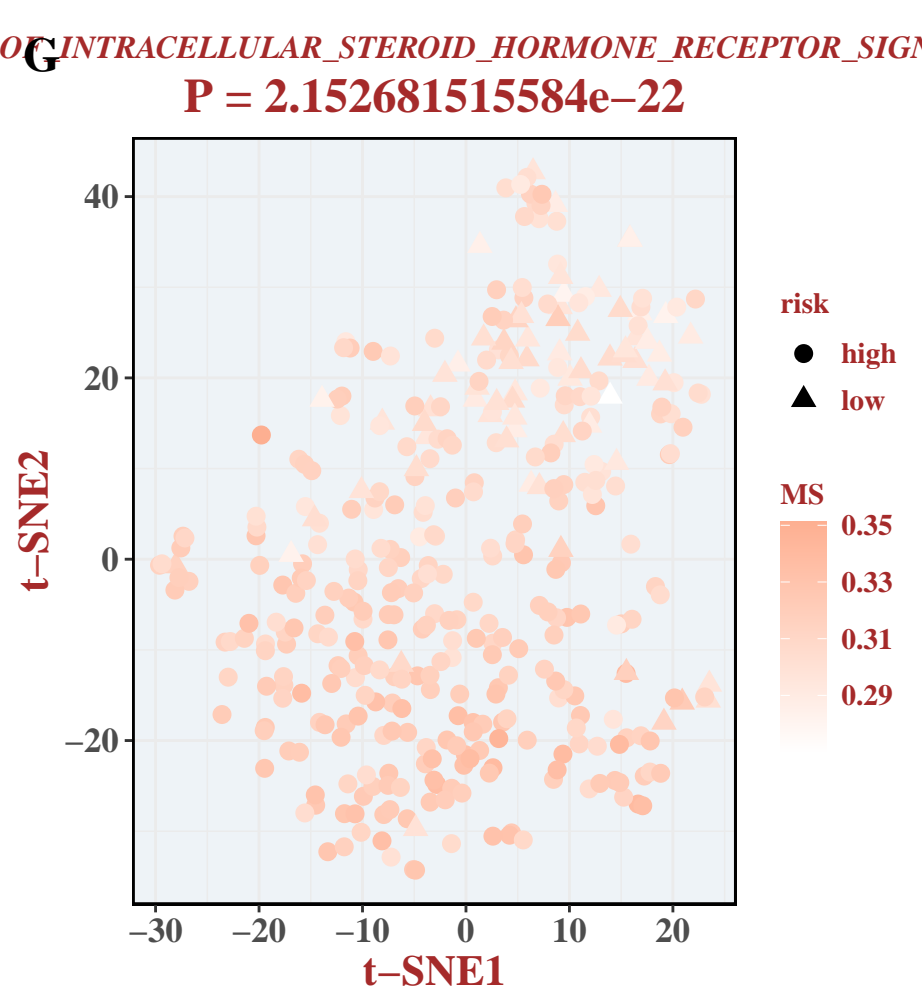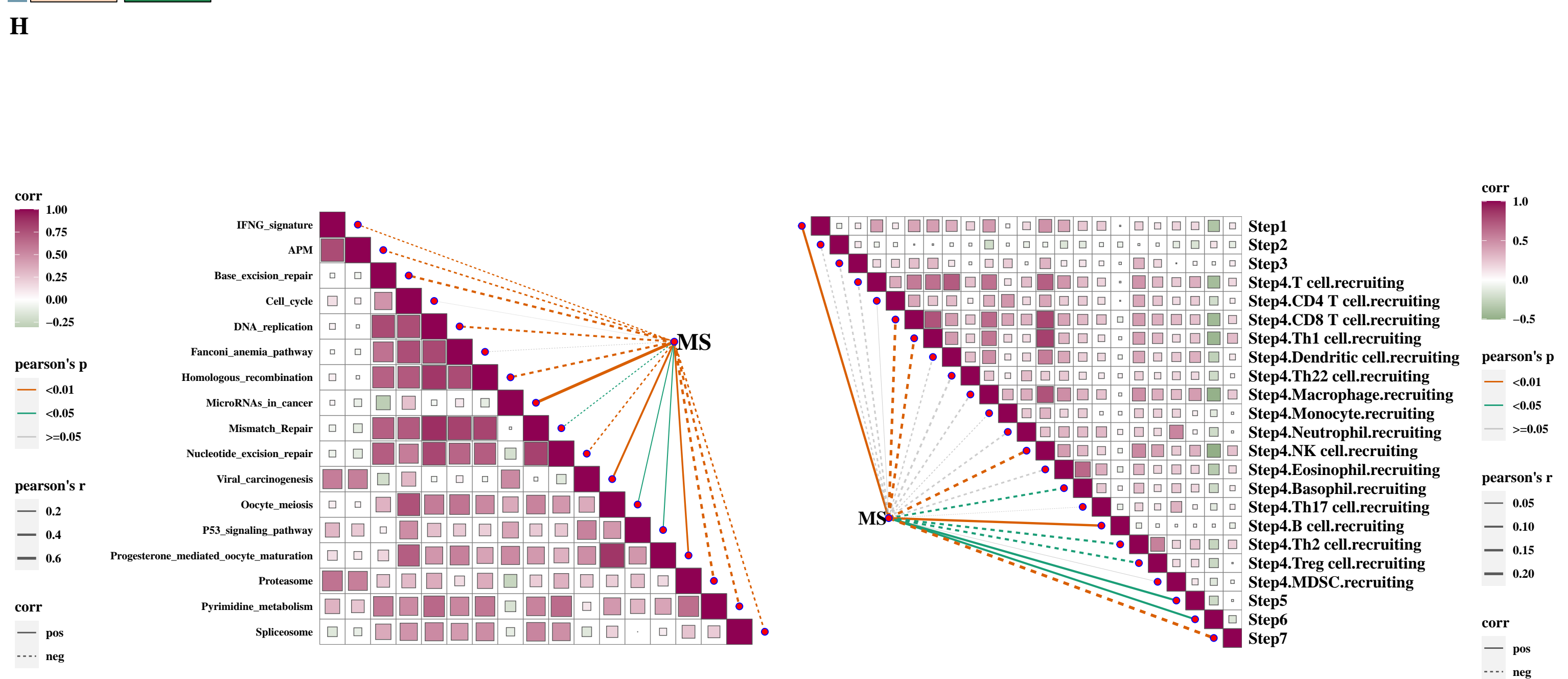

Supplement: Supplementary file 2 — Supplementary material 2: Suppl Figure 2. A-G. Functional analysis of Msigdb gene sets between high and low MS groups. H. Correlation analysis of MS model with TIP tumor immunity and immunotherapy pathways. [file 12672_2025_2892_MOESM2_ESM.pdf]

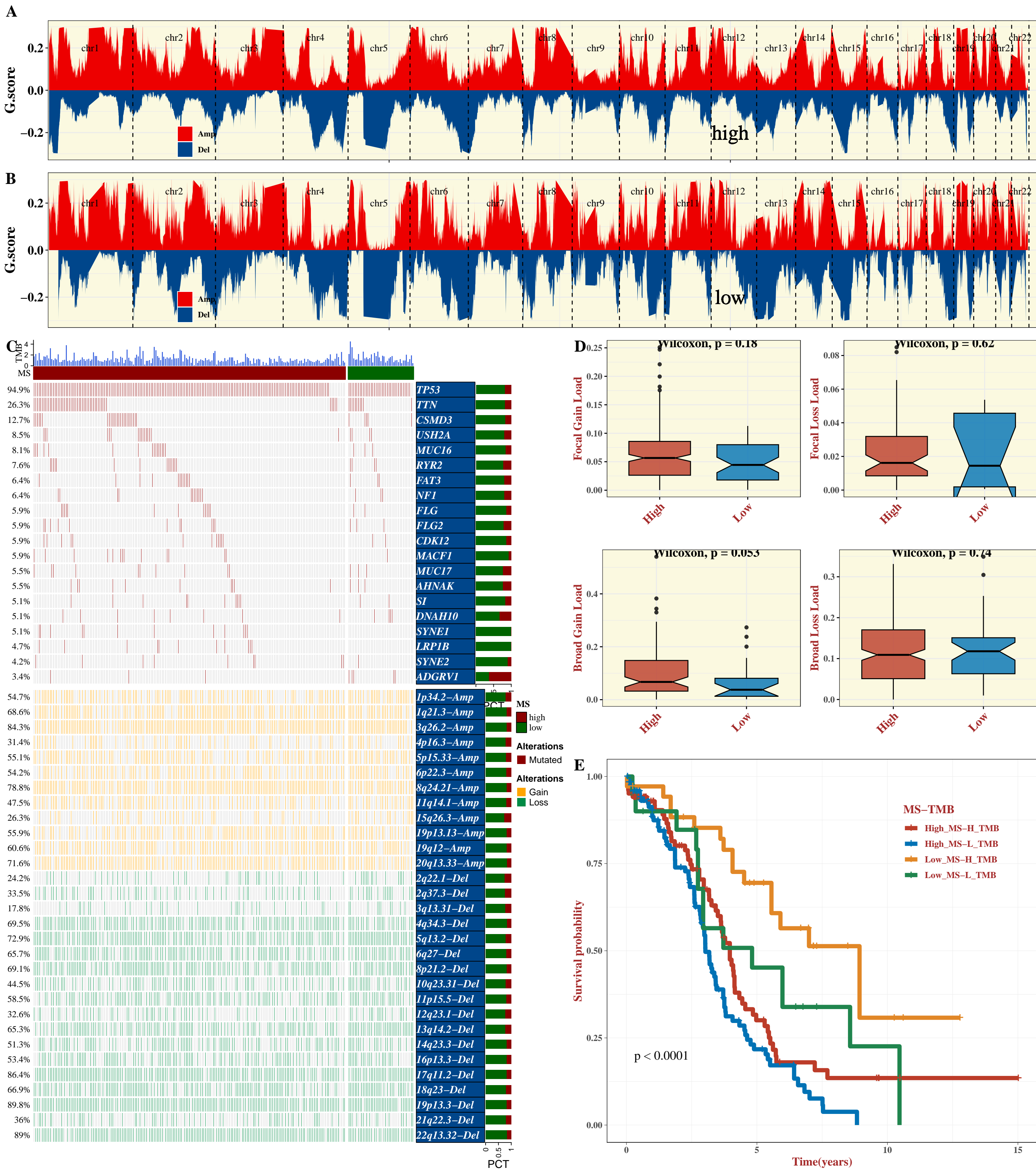

Supplement: Supplementary file 3 — Supplementary material 3: Suppl Figure 3. A-B. CNV results obtained from the gistic2.0 software in high and low MS groups. C. The mutation results based on TCGA mutation data calculated by maftools software. D. The difference between high and low MS groups in Broad and Focal aspects of the CNV results. E. K-M curves of the TMB values combined with MS score in different groups. [file 12672_2025_2892_MOESM3_ESM.pdf]

Outgoing signaling patterns – MS\_high

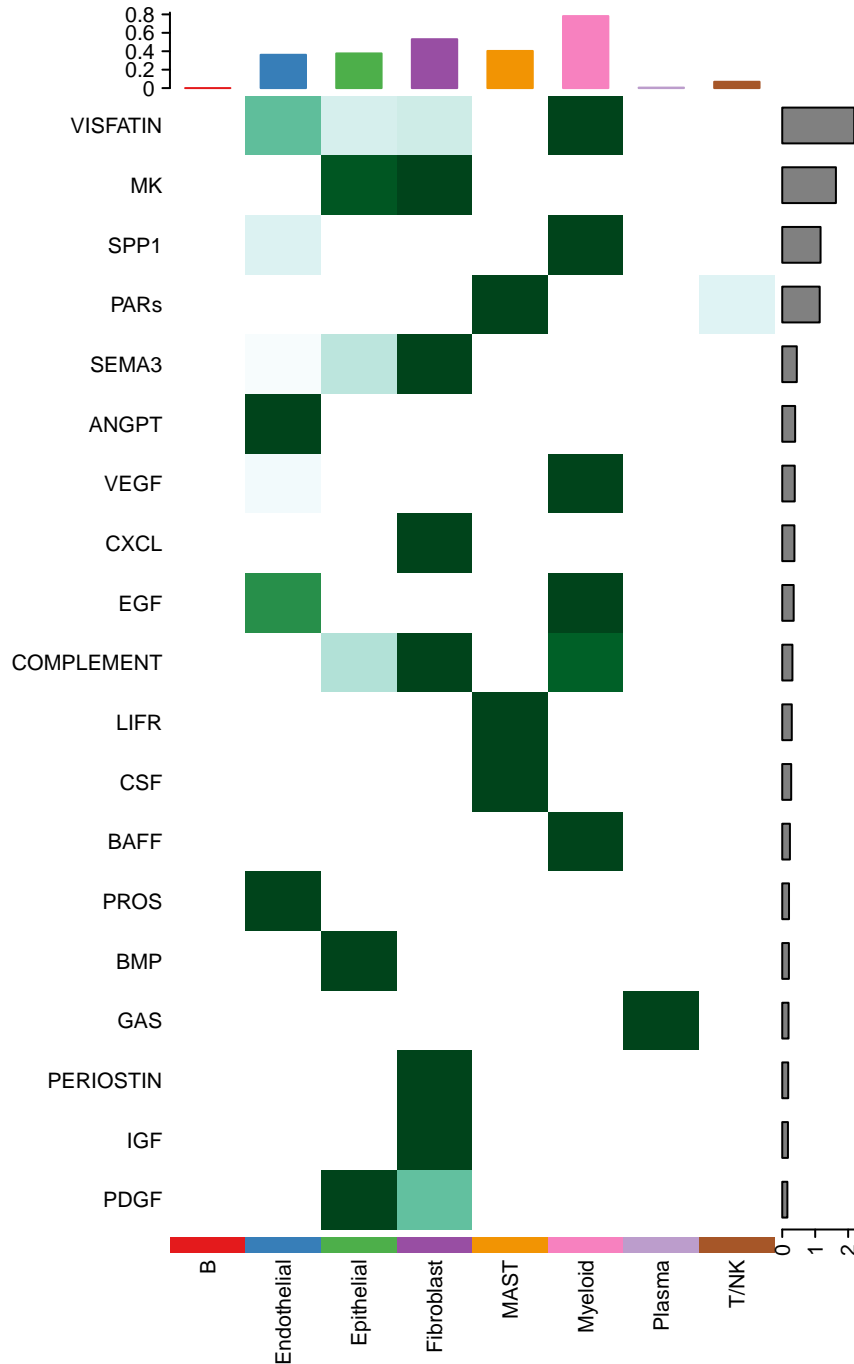

Outgoing signaling patterns – MS\_low

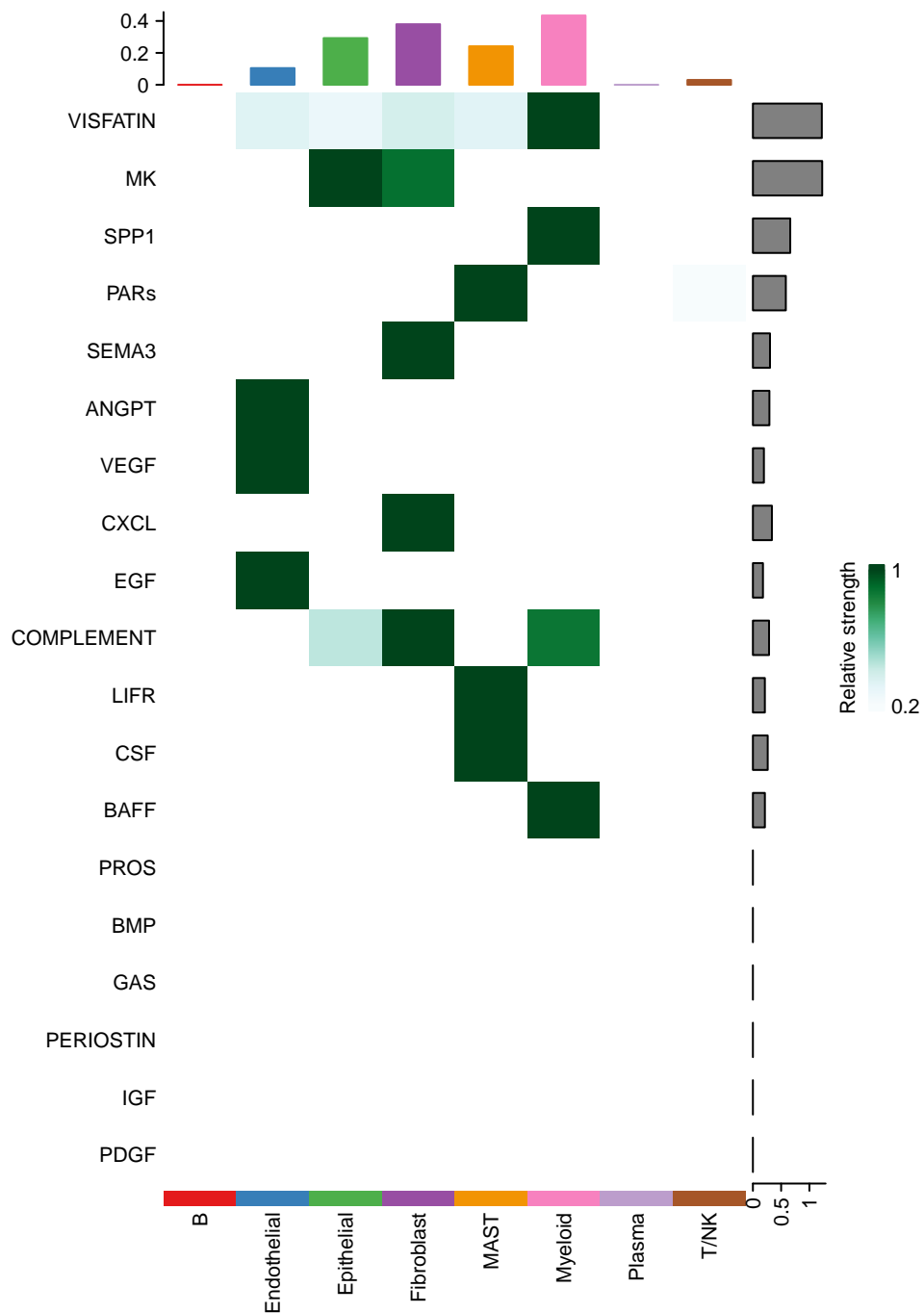

Supplement: Supplementary file 4 — Supplementary material 4: Suppl Figure 4. Heat maps of communication between high and low MS groups in single cell data. [file 12672_2025_2892_MOESM4_ESM.pdf]

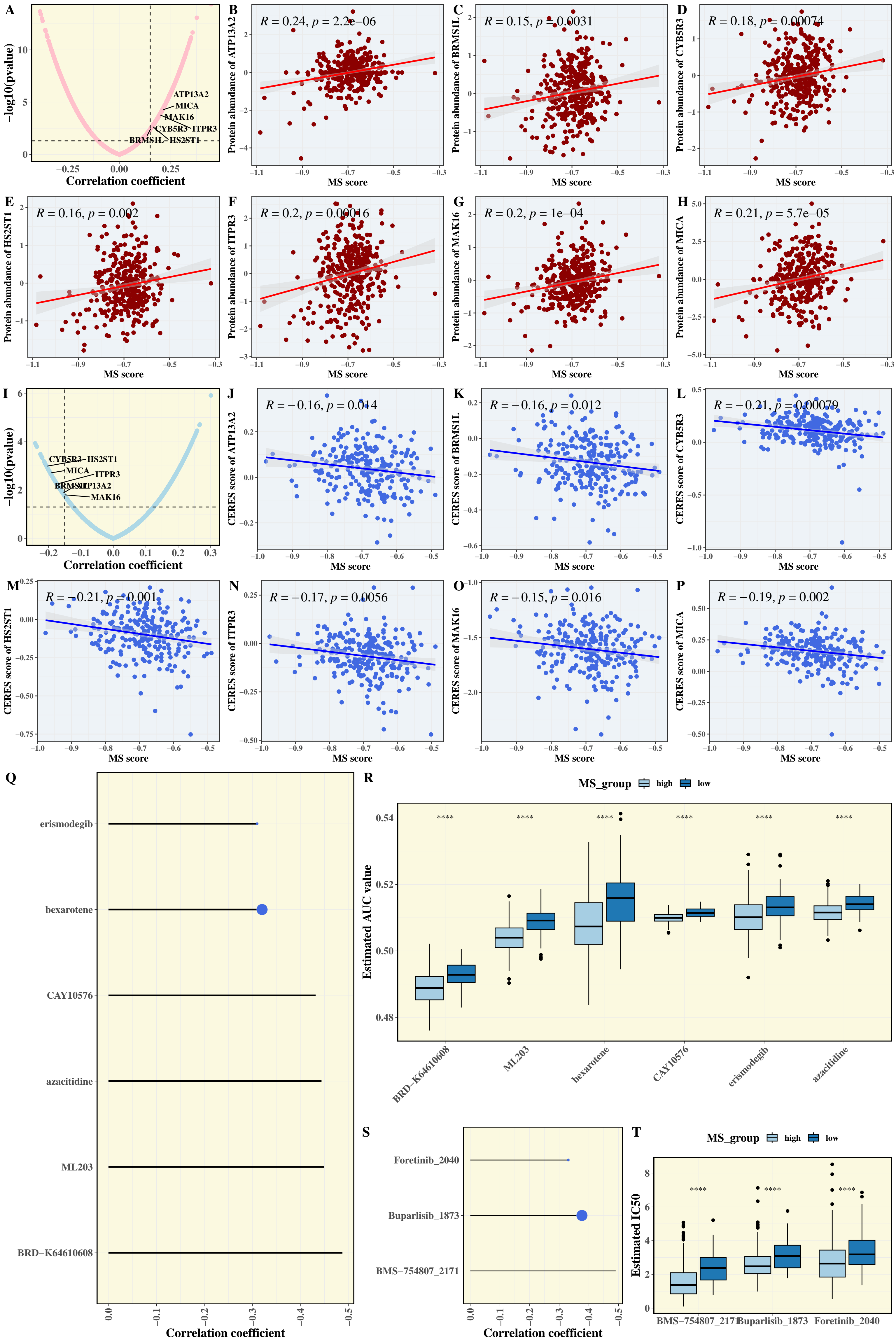

Supplement: Supplementary file 5 — Supplementary material 5: Suppl Figure 5. A-H. Correlation between MS and protein abundance. I-P. The relevance of MS to CERES. Q-T. Relevance of MS to drugs from GDSCv2 and CTRP databases. [file 12672_2025_2892_MOESM5_ESM.pdf]
